# Management of children with obesity at local hospital and impact of COVID-19 pandemic

**DOI:** 10.3389/fped.2023.1228681

**Published:** 2023-08-10

**Authors:** Takeshi Ninchoji, Yuya Aoto, Natsuki Momo, Jun Maruyama, Hiroaki Ioi, Hayato Uchida

**Affiliations:** ^1^ Department of Paediatrics, Harima Himeji General Medical Centre, Himeji, Japan; ^2^ Ioi Pediatrics Clinic, Himeji, Japan; ^3^ School of Human Science and Environment, University of Hyogo, Himeji, Japan

**Keywords:** obesity screening, COVID-19, obesity index, body mass index, children

## Abstract

This study investigated the status of children with obesity before and after the COVID-19 pandemic, and the effects of lifestyle guidance on weight loss among children in Japan. We analysed the data of patients who visited our hospital after check-ups for obesity and evaluated the efficacy of lifestyle guidance. The patients were divided into groups A, B, and C (year 2011, 2019, and 2021, respectively). There were no differences in body weight, obesity index (OI), blood pressure, or alanine transaminase (ALT) levels between the groups; however, aspartate transaminase (AST) level was the highest in Group C. In Group C, only OI increased between the primary and secondary screenings; however, OI and body mass index (BMI) improved during the second screening and more children in the weight loss group followed lifestyle guidance. OI/BMI did not change over the past decade; however, short-term weight gain was significant owing to the COVID-19 pandemic, and simple guidance was effective in reducing weight. Future challenges include identifying methods to achieve long-term weight loss.

## Introduction

Obesity in childhood is associated with a high risk of cardiovascular diseases in adulthood. For several decades, obesity screening has been conducted among elementary school students in Himeji City, Japan. The screening system consists of two steps: blood testing and easy guidance at the primary care clinic. Patients with abnormal results or severe obesity were included in the second stage of the study. They visited to hospitals to undergo blood tests and detailed nutritional and lifestyle guidance.

The coronavirus disease (COVID-19) pandemic, which began at the end of 2019, has affected the world in several ways. With the spread of the virus, lockdowns forced people in many countries to stay indoors. Changes in lifestyle during the COVID-19 pandemic affected obesity in children, as indicated by a few reports on this from countries other than Japan (1, 2). In Japan, staying home was recommended by the ordinances to prevent the spread of the disease; however, no lockdown was established. In this study, we addressed the status of obesity screening before and after the COVID-19 pandemic and resolved the problems, while comparing the results with those from 10 years ago.

## Materials and methods

The patients were elementary school students with obesity living in Himeji City who visited our hospital in 2011 (Group A; 10 years ago), 2019 (Group B; before the pandemic), and 2021 (Group C; during the pandemic) for secondary medical examinations.Obesity index (OI) was calculated using the following equation: OI = [(real body weight)−(standard weight)]/(standard weight)X100(%). In Japan obesity was defined as OI ≥ 20% in children. The standard weight was based on an investigation conducted by the Japanese Ministry of Education, Culture, Sports, Science and Technology in 2000. The criteria of the screening system were as follows:

• School checkup: Children with ≥20% OI were eligible for the primary medical check-up.
• Primary medical checkups: Measurement of OI, body mass index (BMI), blood pressure (BP), and blood tests (aspartate transaminase [AST], alanine transaminase [ALT], total cholesterol [Tchol], low-density lipoprotein cholesterol [LDL], triglyceride [TG], blood sugar [BS]) were performed at the clinic. Any two subjects with an OI ≥ 50%, systolic BP ≥ 135 mmHg, AST/ALT ≥ 50 IU/L, Tchol ≥ 220 mg/dl, LDL ≥ 140 mg/dl, TG ≥ 250 mg/dl, and BS ≥ 126 mg/dl were eligible for secondary medical examination. The period from school to primary examinations is usually approximately three to four months.
• Secondary medical examinations: OI, BMI, BP, and blood tests were conducted. Blood tests included AST, ALT, Tchol, LDL, high-density lipoprotein cholesterol (HDL), TG, uric acid (UA), BS, and thyroid hormone levels. The time period between the primary and secondary examination is approximately three to five months.

Patients in Group C were guided on weight loss by a doctor in our hospital. In groups A and B, only a piece of paper describing precautions against obesity was provided. Nutritional guidance was provided to students with an OI ≥ 35% in all groups. The patients were evaluated one month later to analysed the results of the lifestyle guidance for weight loss. The following five guidelines, which were partially modified from the report by Uchida et al. (3), were emphasized by a doctor when discussing weight loss guidance in Group C:

(1) To drink only water or sugar free tea. Beverages were allowed to be consumed once per week.
(2) To eat every meal with family.
(3) Food is served for each dish.
(4) To exercise for least 15 min a day, twice a week (excluding gyms and commuting time).
(5) To reduce game or screen times to minus 1 h.

Each data point was examined and statistically analysed using JMP 10.0 (JMP Statistical Discovery LLC, Cary, NC, United States). Data are expressed as median values with range and the Shapiro-Wilk test was used to confirm the lack of Gaussian distribution in each data set. Groups were compared using the Mann-Whitney U or Wilcoxon' s paired-order test. Qualitative data were analysed using Fisher's exact test. Statistical significance was set at *p* < 0.05. This study was approved by the ethics committee of Steel Memorial Hirohata Hospital (#302, approved at 17th of November, 2021, the hospital name was changed to Harima-Himeji General Medical Hospital in May, 2022).

## Results

Table 1 presents the results of the study. There were 30, 27, and 45 patients in Groups A, B, and C, respectively. There were no significant differences in age or sex between the groups. Moreover, during the primary medical examination at the clinic, there were no significant differences in height, weight, BMI, or OI among the groups. Additionally, there were no significant differences in systolic BP, and levels of ALT, Tchol, and TG levels between the groups. Although the median AST levels were within the normal range, Group C had the highest values, with a significant difference. Group B showed low LDL levels, whereas groups A and C did not show any significant changes. BS levels were also within the normal range but increased annually. Upon second medical examination at our hospital, there was no significant difference in all parameters except for the BS level; however, in group C, only OI but not BMI/BMI z-score notably increased from the first checkup, revealing that only OI increased within a few months. In the second round of medical examinations, the tendency was the same as that in the first checkup; however, the changes in OI and BMI (not BMI z-score) due to guidance was the highest in Group C, and weight reduction in one month was apparent. Finally, we examined the specific content of the instructions and compliance with achievement in Group C. The weight loss success group clearly adhered to the following instructions more than the unsuccessful group: “(1) drink water or sugar free tea, drink juice less than once” and “(4) exercise for 15 min or more a day, at least twice a week (excluding physical education and commuting time)”, and “(5) game and screen time of 1 h” (see Table 2).

**Table 1:** Results of physical and blood examination at primary and secondary medical check-up.

	Group A	Group B	Group C	Total	*p*
Total	30	27	45	102	
Age	10.6 (10.0–11.2)	11.3 (10.8–11.8)	11.0 (10.5–11.4)	11 (10.7–11.2)	0.28
M/F	8/22	10/17	13/32	31/71	0.67
Primary medical check-up					
Height	136.2 (132.6–139.8)	140.6 (135.9–145.3)	139.0 (136.2–141.7)	138.6 (136.6–140.6)	0.32
Height z-score	1.0 (0.5–1.4)	1.2 (0.2+−2.6)	1.1 (0.7–1.9)	1.1 (0.6–1.9)	0.57
Weight	43.4 (38.7–48.1)	49.6 (43.1–56.2)	47.3 (44.3–50.3)	46.8 (44.2–49.2)	0.45
Weight z-score	2.6 (2.1–3.4)	2.5 (1.7–4.5)	3.0 (1.9–4.1)	2.7 (2.0–4.0)	0.72
BMI	23.7 (17.2–32.3)	23.9 (19.0–35.1)	23.8 (18.1–32.8)	23.8 (17.2–35.1)	0.90
BMI z-score	1.9 (1.8–2.3)	1.9 (1.5–2.4)	2.0 (1.7–2.4)	2.0 (1.7–2.4)	0.89
Obesity index	40.5 (35.1–45.9)	39.1 (32.1–46.0)	39 (33.8–44.3)	39.5 (36.2–42.7)	0.71
Systolic BP	115.4 (110.6–120.1)	113.9 (106.7–121.0)	109.8 (103.7–115.9)	112.5 (109.0–115.9)	0.44
AST	31.6 (27.2–36.1)	30.7 (24.2–37.1)	38.5 (34.0–43.1)	34.4 (31.5–37.4)	<0.01
ALT	38.7 (29.8–47.6)	37.4 (21.1–53.8)	48.4 (38.0–58.9)	42.7 (36.0–49.4)	0.13
Tchol	196.6 (184.0–209.3)	185.9 (170.1–201.6)	204.8 (196.1–213.5)	197.4 (190.7–204.1)	0.18
LDL	123.2 (112.9–133.6)	109.8 (97.6–122.0)	128.7 (120.7–136.6)	122.1 (116.4–127.7)	<0.01
TG	107.9 (90.5–125.3)	135.7 (105.9–165.5)	128.2 (105.9–150.4)	124.2 (110.9–137.5)	0.50
BS	88 (86.1–89.9)	90.9 (88.0–93.7)	91.1 (89.6–92.6)	90.1 (89.0–91.3)	<0.05
Secondary medical check-up (1st Visit)					
Height	139.1 (135.8–142.6)	142 (137.3–146.7)	139.8 (137.0–142.6)	140.2 (138.2–142.1)	0.66
Height z-score	1.4 (0.6–1.8)	1.1 (0.2–2.4)	1.0 (0.5–1.7)	1.1 (0.5–1.9)	0.73
Weight	47.5 (43.8–51.2)	50 (43.4–56.6)	48.2 (45.0–51.4)	48.5 (46.1–50.9)	0.96
Weight z-score	2.8 (2.0–3.8)	2.2 (1.4–4.6)	2.8 (1.7–3.8)	2.8 (1.8–4.0)	0.69
BMI	39.2 (33.1–51.2)	39.3 (32.9–45.7)	42.8 (37.0–48.6)	40.8 (37.4–44.3)	0.66
BMI z-score	2.0 (1.7–2.3)	1.8 (1.6–2.4)	2.0 (1.6–2.4)	2.0 (1.6–2.3)	0.70
Obesity index	24.3 (16.9–32.5)	23.2 (15.5–33.8)	23.8 (17.0–33.4)	23.8 (15.5–33.8)	0.87
Systolic BP	104.5 (99.1–109.8)	108.2 (103.–113.0)	107.5 (104.0–111.0)	106.8 (104.3–109.3)	0.81
AST	28 (24.9–31.1)	26.6 (22.1–31.0)	32.3 (26.6–38.0)	29.5 (26.6–32.4)	0.18
ALT	27.9 (20.3–35.5)	26.8 (19.2–34.5)	41.4 (25.9–57.0)	33.6 (26.2–41.0)	0.29
Tchol	197.9 (186.1–209.8)	181.6 (168.1–195.2)	195 (184.9–205.0)	192.3 (185.7–198.9)	0.19
LDL	124.2 (114.3–134.1)	113.5 (103.1–124.0)	123.8 (114.6–133.1)	121.2 (115.6–126.8)	0.31
HDL	58.8 (54.2–63.4)	55.1 (49.3–60.9)	52.8 (49.0–56.7)	55.2 (52.6–57.8)	0.14
TG	76 (61.1–90.9)	81.2 (66.3–96.2)	96.4 (76.3–116.5)	86.4 (75.9–96.9)	0.56
BS	87.6 (84.3–90.9)	93.4 (91.2–95.6)	94.2 (92.4–95.9)	92.0 (90.6–93.5)	<0.001
UA	5.2 (4.9–5.6)	4.9 (4.4–5.4)	5.2 (5.0–5.7)	5.2 (5.0–5.4)	0.36
Change BMI	−0.002 (−1.7–−4.6)	0.27 (−3.9–−2.1)	0.32 (−1.9–−2.1)	0.23 (−3.9–−4.6)	0.59
Change BMI z-score	0.04 (−0.1–0.2)	0.001 (−0.08–0.2)	0.005 (−0.07–0.1)	0.006 (−0.08–0.15)	0.90
Change OI	−1.3 (−3.6–1.0)	0.2 (−3.3–3.8)	3.8 (2.2–5.3)	1.3 (0.0–2.7)	<0.01
Secondary medical check-up (2st Visit)					
Height	139.8 (136.4–143.3)	142.5 (137.9–147.1)	140.3 (137.5–143.1)	140.7 (138.8–142.7)	0.72
Height z-score	1.1 (0.4–1.5)	0.8 (−0.1–1.9)	0.8 (0.3–1.6)	0.8 (0.2–1.6)	0.69
Weight	47.9 (44.1–51.6)	50.6 (43.8–57.4)	48.4 (45.0–51.8)	48.8 (46.3–51.3)	0.99
Weight z-score	2.4 (1.9–3.7)	2.1 (1.0–4.2)	2.4 (1.5–3.4)	2.4 (1.5–3.6)	0.69
BMI	24. (20.3–32.3)	23.2 (15.4–35.4)	23.8 (17.5–39.3)	23.8 (15.5–39.3)	0.90
BMI z-score	2.0 (1.7–2.2)	1.8 (1.5–2.4)	2.0 (1.5–2.3)	1.9 (1.5–2.3)	0.69
OI	38.6 (32.3–44.9)	39.1 (32.3–45.9)	40.7 (35.1–46.3)	39.7 (36.2–43.1)	0.89
Systolic BP	113.2 (109.1–117.4)	107.9 (103.8–112.1)	107.5 (103.4–111.5)	109.3 (106.9–111.7)	0.14
AST	26.1 (24.4–27.8)	24.8 (21.5–28.0)	30 (24.7–35.3)	27.5 (25.0–30.0)	0.15
ALT	25.5 (20.3–30.7)	25.4 (17.0–33.8)	36.7 (23.1–50.4)	30.4 (23.9–36.9)	0.17
Tchol	198.9 (188.1–209.6)	185.3 (172.0–198.6)	191.7 (180.3–203.1)	192.1 (185.4–198.8)	0.27
LDL	122.7 (113.6–131.7)	115.4 (104.6–126.2)	120.8 (111.0–130.5)	119.9 (114.2–125.6)	0.50
HDL	58.8 (53.6–64.0)	57.7 (51.0–64.4)	53.8 (50.2–57.5)	56.3 (53.6–59.1)	0.42
TG	83.5 (67.4–99.6)	79.8 (65.8–93.7)	90 (70.6–109.4)	85.4 (75.2–95.6)	0.95
UA	5.2 (4.7–5.6)	5.0 (4.4–5.5)	5.3 (4.9–5.6)	5.1 (4.9–5.4)	0.38
BS	87.4 (84.9–89.9)	92.7 (90.2–95.1)	93.7 (91.8–95.6)	91.6 (90.2–93.0)	<0.001
Change BMI	−0.063 (−1.13–−1.20)	0.019 (−0.89–−1.63)	0.302 (−1.47–−9.39)	−0.09 (−1.47–−9.39)	<0.05
Change BMI z-score	0.05 (−0.015–0.11)	0.031 (−0.01–0.1)	0.09 (0.035–0.15)	0.04 (−0.01–0.15)	0.05
Change OI	−0.597 (−1.97–0.77)	−0.185 (−1.48–1.11)	−2.13 (−3.1–−1.2)	−1.16 (−1.84–−0.49)	<0.05

**Table 2:** Compliance rate of guidance in successful and unsuccessful weight loss.

	Successful weight loss	Unsuccessful weight loss	Unchanged	Total
Compliance for No. 1	15/16 (94%)	2/3 (67%)	5/6 (83%)	22/25 (88%)
Compliance for No. 2	0/0 (0%)	0/1 (0%)	0/0 (0%)	0/1 (0%)
Compliance for No. 3	5/5 (100%)	0/0 (0%)	1/2 (50%)	6/7 (86%)
Compliance for No. 4	12/16 (75%)	1/4 (25%)	4/7 (57%)	17/27 (63%)
Compliance for No. 5	10/25 (40%)	1/5 (20%)	0/7 (0%)	11/37 (30%)

## Discussion

Although guideline regarding obesity in children are few in Japan, several studies indicated that 40% of school-age obesity leads to a high prevalence of adult obesity (4–6), which is associated with an increase in lifestyle-related diseases and mortality (7, 8). The number of children with obesity continued to increase in the 1900s but the prevalence has plateaued since 2006, in Japan. However, the pandemic of COVID19 infection and restrictions on movement have had a major impact on children in Japan, and obesity has increased because they cannot exercise or go outside (9). The 2020 School Health Statistics report indicated that the percentage of children with obesity has increased in each age group; additionally snack consumption has increased and people are eating well-balanced meals during pandemic (10). This may be due to the decreasing proportion of households with less sleep and more screen time (1). As this study was a secondary medical examination, the data cannot be used to verify changes in obesity and the increase in the prevalence of obesity. The increase in the number of patients visiting our hospital may reflect the increase in obesity. In the future, we shall analyse this by researching on the entire Himeji city. In addition, the values of AST, ALT, TG, and BS levels were within the normal ranges; the differences in these values before and after the COVID-19 pandemic were only slight, suggesting that their association with obesity is unclear. However, changes in OI (but not BMI) in Group C (=during the pandemic) over a short period of time were possibly related to the pandemic. Lifestyle and eating behaviours should also be investigated. However, the guidance was effective among students during the pandemic.

Referring to the guidance, at our hospital, we slightly modified the report of Uchida et al. (3) Initially, previous reports indicated that the intake of sugary juices is closely related to obesity (11). Overall, 22/25 (88%) outpatients were able to follow the guidance, which implies its ease of implementation. Regarding exercise therapy, implementing concrete plans may have been challenging due to the pandemic, and the overall adherence rate remained at 67%. However, there was a clear difference between the success and failure of weigh loss. Finally, the screen time of children was restricted. However, only 30% of the patients actually adhered to this, and 40% of them successfully lost weight. The fact that we were forced to live indoors owing to the pandemic may have influenced adherence to screen time restriction; however, undoubtedly, compared to 10 years ago, our lifestyles increasingly involve the use of computers or games. In addition, the pandemic has accelerated it. In the past, it was reported that obesity decreased by nearly 40% with less than 2 h of television time (12), and screen time is likely to be a major issue in the future. Thus, short-term weight gain was significant owing to pandemic, and conversely, simple guidance was effective in reducing weight, at least during the COVID pandemic.

## Limitations

First, it was a very small study that only summarized data from our hospital. There are only preliminary data, as we are planning to use the entire Himeji City in the near future. Second, healthcare guidance was implemented only during the pandemic (Group C), and an analysis of its effect and its association with the pandemic was not possible. Third, lifestyle and eating behaviour were not investigated in this study.

## Conclusions

During the pandemic, only OI increased between the primary and secondary screenings. However, OI and BMI improved during the second screening and more children in the weight loss group followed lifestyle guidance. Simple guidance may be effective in reducing weight. Future large-scale studies should reveal methods to achieve long-term weight loss.

## Data Availability

The original contributions presented in the study are included in the article/Supplementary Material, further inquiries can be directed to the corresponding author.
